# Heterologous Replacement of the Supposed Host Determining Region of Avihepadnaviruses: High In Vivo Infectivity Despite Low Infectivity for Hepatocytes

**DOI:** 10.1371/journal.ppat.1000230

**Published:** 2008-12-05

**Authors:** Kai Dallmeier, Ursula Schultz, Michael Nassal

**Affiliations:** 1 University Hospital Freiburg, Internal Medicine II/Molecular Biology, Freiburg, Germany; 2 Cellgenix, Freiburg, Germany; University of California San Francisco, United States of America

## Abstract

Hepadnaviruses, including hepatitis B virus (HBV), a highly relevant human pathogen, are small enveloped DNA viruses that replicate via reverse transcription. All hepadnaviruses display a narrow tissue and host tropism. For HBV, this restricts efficient experimental in vivo infection to chimpanzees. While the cellular factors mediating infection are largely unknown, the large viral envelope protein (L) plays a pivotal role for infectivity. Furthermore, certain segments of the PreS domain of L from duck HBV (DHBV) enhanced infectivity for cultured duck hepatocytes of pseudotyped heron HBV (HHBV), a virus unable to infect ducks in vivo. This implied a crucial role for the PreS sequence from amino acid 22 to 90 in the duck tropism of DHBV. Reasoning that reciprocal replacements would reduce infectivity for ducks, we generated spreading-competent chimeric DHBVs with L proteins in which segments 22–90 (Du-He4) or its subsegments 22–37 and 37–90 (Du-He2, Du-He3) are derived from HHBV. Infectivity for duck hepatocytes of Du-He4 and Du-He3, though not Du-He2, was indeed clearly reduced compared to wild-type DHBV. Surprisingly, however, in ducks even Du-He4 caused high-titered, persistent, horizontally and vertically transmissable infections, with kinetics of viral spread similar to those of DHBV when inoculated at doses of 10^8^ viral genome equivalents (vge) per animal. Low-dose infections down to 300 vge per duck did not reveal a significant reduction in specific infectivity of the chimera. Hence, sequence alterations in PreS that limited infectivity in vitro did not do so in vivo. These data reveal a much more complex correlation between PreS sequence and host specificity than might have been anticipated; more generally, they question the value of cultured hepatocytes for reliably predicting in vivo infectivity of avian and, by inference, mammalian hepadnaviruses, with potential implications for the risk assessment of vaccine and drug resistant HBV variants.

## Author Summary

Hepatitis B virus (HBV) associated liver disease is a leading cause of death worldwide. Host range restrictions limit experimental HBV infections largely to chimpanzees or isolated human hepatocytes. A narrow host range is shared by the animal hepadnaviruses, e.g. from ducks (DHBV) and herons (HHBV); HHBV does not infect ducks though it can establish a low-level infection in cultured duck hepatocytes. Host tropism is thought to be mediated by the PreS domain of the large viral envelope protein, because certain duck virus PreS segments introduced into the envelope of spreading-incompetent HHBV pseudotypes enhanced infectivity for duck hepatocytes. Expecting that reciprocal exchanges in DHBV would negatively impact duck tropism, we generated chimeric DHBVs in which the PreS regions in question are derived from HHBV and which are autonomously spreading-competent; this allowed us to directly compare their infectivity for duck hepatocytes and ducks. Surprisingly, even the chimera with the largest portion of HHBV sequence was as infectious for ducks as authentic DHBV; in vitro infectivity, however, was substantially reduced. These unexpected differences question the value of cultured hepatocytes to reliably predict in vivo infectivity of avihepadnaviruses and, by inference, also that of vaccine escape and therapy resistant HBV variants.

## Introduction

Hepadnaviruses including hepatitis B virus (HBV), the causative agent of B-type hepatitis in humans, are small enveloped hepatotropic DNA viruses that replicate by reverse transcription ([1]; for reviews see [2],[3]). Related viruses have been found in some mammals (orthohepadnaviruses), e.g. woodchucks (WHV), and in selected bird species (avihepadnaviruses), e.g. ducks (DHBV) and herons (HHBV). In general, each of these viruses can infect only a limited range of hosts [4],[5].

**Figure 1:**
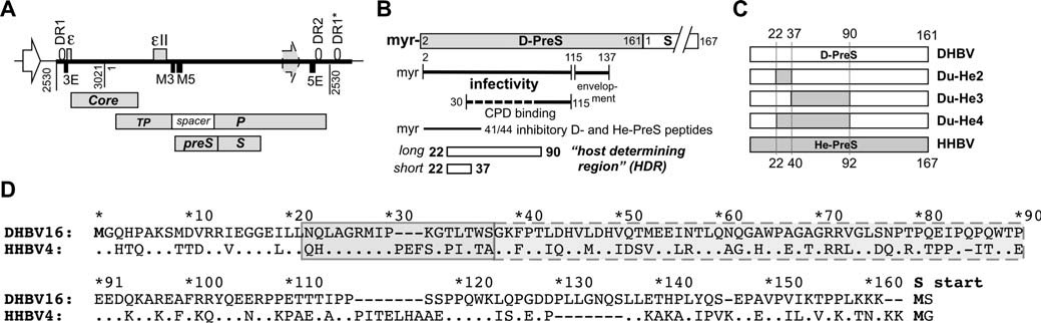
Hepadnavirus genome organization and functional PreS domains. (A) DHBV genome. The black line represents the vector-borne 1.1× genome driven by a heterologous promoter; the grey arrow symbolizes the viral core promoter controlling pgRNA expression on cccDNA. Numbers are nucleotide positions. Indicated cis-elements: DR, direct repeat; ε and εII, RNA encapsidation signals; E and M, required for RC-DNA formation. Bars represent the indicated ORFs; TP, Terminal Protein domain of P. (B) Functional regions in DHBV PreS. Numbers are amino acid positions; myr, myristic acid. The carboxypeptidase D (CPD) binding region [50] contains an essential (solid line) and an auxiliary part (dashed extension). The long and short forms of the proposed host-determining region (HDR) are denoted by white bars. (C) Chimeric PreS regions used. Segments from D-PreS and He-PreS present in the chimeric viruses are indicated. (D) DHBV versus HHBV PreS. The PreS amino acid sequences of DHBV16 [70] and HHBV4 [24] are shown as aligned by the Clustal×algorithm. The proposed HDR forms are boxed. Numbers are amino acid positions for D-PreS. Dots, identical amino acids; dashes, gaps.

Nonetheless, all hepadnaviruses share a similar genome organization and replication strategy. Upon infection the about 3 kb relaxed circular (RC) DNA genome in virions is converted into covalently closed circular (ccc) DNA that acts as transcription template. The greater-than-genome length pregenomic (pg) RNA serves as mRNA for the capsid protein and the reverse transcriptase (P protein), and via specific P-RNA interactions is encapsidated and reverse transcribed into progeny RC-DNA (for review, see [6]). New nucleocapsids can either recycle the RC-DNA to the nucleus for cccDNA amplification, or be secreted after envelopment by the surface, or envelope, proteins (for review, see [7]) that are translated from subgenomic RNAs. N terminal addition to the small surface protein (S), a transmembrane protein, of the PreS1 plus PreS2 domains in orthohepadnaviruses, or of a single about 160 amino acid (aa) PreS domain in avihepadnaviruses, creates the respective large envelope proteins (L). PreS2 plus S form the middle (M) protein in the mammalian viruses. Notably, the *preS/S* open reading frames (ORFs) overlap entirely with the *P* ORF (Figure 1A).

Hepatotropism is largely attributable to the requirement for hepatocyte-enriched transcription factors [8]; host range appears to be mainly controlled at the early steps of virus attachment and entry as inferred from the ability of several non-infectable cell lines, even of heterologous species origin [9], to produce infectious virions when transfected with cloned hepadnaviral DNA. Infection, however, usually requires primary hepatocytes. Because none of the natural hosts is an established experimental animal, few different species have been investigated; these include hepatocytes from humans and tupaias [10],[11], or woodchucks (reviewed in [12]); Pekin ducks (*Anas platyrhynchos var. domestica*) provide the only feasible source for hepatocytes of a genuine avihepadnavirus host (reviewed in [13]). Even greater restrictions apply to in vivo experiments. Collectively the few published studies suggest the existence of a species barrier that prevents efficient transmission to other than closely related hosts. HBV can be transmitted to chimpanzees [14] but not [15], or only extremely inefficiently [16], to baboons; WHV was not infectious for ground squirrels [17]; and DHBV does not infect chicken [18]. Notably, though, this barrier is not absolute; for instance, Beechey Ground Squirrel HBV (GSHV) was infectious for woodchucks and chipmunks [19], though not mice or rats [20]. In vitro, host tropism appears more relaxed as shown by the relatively efficient infectibility of tupaia hepatocytes [10],[11] by HBV and woolly monkey HBV (WMHBV; [21]), or of duck hepatocytes by crane hepatitis B virus [22]. While these examples attest to an as yet poorly understood complexity of hepadnaviral host tropism (reviewed in [5]), the inability of HHBV to establish detectable infection in ducks is solidly established [23],[24].

Though numerous cellular HBV binding proteins have been reported (reviewed in [4]), the receptors for orthohepadnaviruses are not known; the major candidate receptor for DHBV, carboxypeptidase D (CPD; formerly gp180 [25]), is ubiquitously expressed and highly conserved between bird species; hence it can explain neither tissue nor host tropism. Glycine decarboxylase was proposed as a co-receptor [26] yet whether its coexpression with CPD renders non-infectable cells susceptible to DHBV infection was not reported. On the virus side, by contrast, a pivotal role of the PreS domains of the L proteins for infection is firmly established (Figure 1B). Anti-PreS antibodies can block HBV [27] and DHBV infection [28]–[31], and many mutations within aa 1 to 75 of HBV PreS1 [32] and aa 2 to 115 [33] of DHBV PreS (D-PreS) abrogate infectivity, as does prevention of PreS myristoylation. In addition, short C proximal PreS regions are essential for nucleocapsid envelopment and hence for infectious virion formation (reviewed in [4],[7]).

The PreS regions display the highest sequence divergence among hepadnaviruses (42% aa identity for DHBV versus HHBV PreS [He-PreS], compared to 84% for the respective S proteins), suggesting that PreS also harbors elements governing host range. In a landmark study, Ishikawa and Ganem [23] laid the foundation for the current model of host-range determinants in hepadnaviruses, using primary duck hepatocytes (PDH) and DHBV as homologous, and HHBV as a heterologous virus. HHBV was capable of establishing a low level infection in PDH (100 to 1,000-fold less efficiently than DHBV). Infectivity of an envelope-deficient (env^−^) HHBV genome increased upon pseudotyping with chimeric HHBV envelope proteins containing the entire D-PreS domain or its segments 1–90 and 22–108, but not 43–161. It was concluded that the common segment 22–90, or possibly its subsegment 22–37 (T. Ishikawa, personal communication), governs the species-specificity of avihepadnaviral infection. In apparent accord, D-PreS peptides comprising aa 2–41 can efficiently block PDH infection by DHBV [34]. Although a corresponding He-PreS peptide was also inhibitory, PreS aa 22–37 (here termed short form), or possibly 22 to 90 (long form; Figure 1B,D), are often referred to as the host-determining region (HDR) of avihepadnaviruses [4],[34],[35]. Similar pseudotype data with L protein chimeras between HBV and WMHBV on human hepatocytes [36] suggested that essentially the same holds for the orthohepadnaviruses, although recent in vitro infection studies [37],[38] using hepatitis delta virus (HDV), an RNA virus that depends on the envelope of HBV to form infectious particles [39], are difficult to reconcile with such a simple model (see Discussion).

Most importantly, the role of the supposed HDRs in a true in vivo hepadnavirus infection, requiring autonomously replicating viruses that inheritably encode the chimeric envelope proteins, has never been assessed.

The aim of this study was to provide such information. We could, indeed, confirm an increased PDH infectivity of HHBV pseudotypes carrying selected D-PreS segments, including aa 22–37. However, reciprocal replacement of this sequence in an autonomous chimeric DHB virus had no negative impact on infectivity for duck hepatocytes or ducks. Most surprisingly, a DHBV chimera in which the entire proposed HDR was replaced by the corresponding heron virus sequence (Du-He4), was poorly infectious for duck hepatocytes yet was nearly as, if not equally infectious for ducks as wild-type DHBV. Thus although the heterologous PreS sequence limited in vitro infectivity it did not do so in vivo. These results question the value of isolated hepatocytes to reliably predict in vivo host range of avian, and by inference, mammalian hepadnaviruses, with consequences for the risk assessment of human HBV variants that seem to inevitably emerge during current nucleos(t)ide analog based therapeutic regimens of chronic hepatitis B; notably, due to the overlapping ORF arrangement, such mutations in P also affect the envelope genes.

## Results

### Experimental design

**Figure 2:**
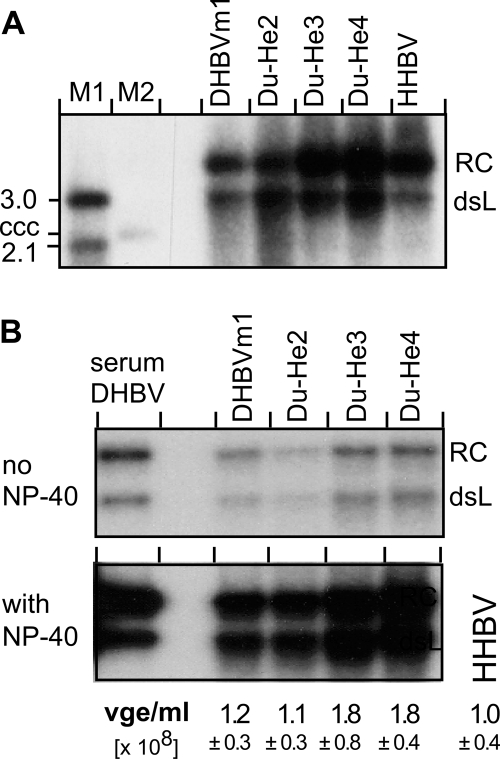
Chimeric viruses are replication-competent and form enveloped virions. (A) Southern blot for intracellular viral DNAs. LMH cells were transfected with expression vectors for the indicated virus genomes, and viral DNAs were detected using a composite DNA probe that recognizes DHBV and HHBV with similar efficiency (see Protocol S1). RC, relaxed circular DNA; dsL, double-stranded linear DNA; M1, dsL DHBV DNA fragments of the indicated sizes (in kb); M2, covalently closed circular (ccc) 3 kb plasmid containing DHBV sequence. (B) Detergent dependence of endogenous polymerase activity. Culture supernatants from the transfected LMH cells were subjected to immunoprecipitation with a monoclonal antibody against D-PreS which also recognizes the chimeric D-PreS proteins but not He-PreS. Serum-derived DHBV served as control. Equal aliquots of the immune pellets were subjected to endogenous polymerase assay conditions in the absence, or in the presence of NP-40 detergent which increased signal intensities, in all samples, by at least 20-fold. Numbers below each lane indicate the concentration of viral genome equivalents (vge) per mL of culture supernatant as determined by qPCR (see Protocol S1); standard deviations are based on four (DHBVm1) or six (all others) independent determinations; residual plasmid DNA accounted for at most one percent of the signals.

Pseudotypes can not spread in vivo. We therefore aimed at introducing the supposedly relevant PreS segment exchanges into autonomous chimeric viruses. If a specific PreS segment determined the duck tropism of DHBV, its replacement by heron virus sequence should decrease infectivity for ducks; the segments replaced in the respective DHBV chimeras were D-PreS 22–37 (Du-He2), 22–90 (Du-He4), and 38–90 (Du-He3), and in an additional chimera not considered here in detail, 91–161 (Du-He7). Concerns for the in vivo experiments were the prevalence of congenital DHBV infections in domestic ducks (10–20% of the ducks used here), and the high in vivo infectivity of DHBV [40] such that even minute contaminations of the inocula might result in wild-type virus infection. For easy distinction, all recombinant viruses, including the DHBV16 (Genbank accession: K01834) derivative DHBVm1 serving as reference wild-type virus, were therefore genetically tagged by unique restriction sites (Figure S1A). PreS segment swapping generates mutant P proteins and could affect genomic cis-elements [6],[41]. However, replication competence and envelopment were not negatively affected in the three chimeras (and neither in Du-He7). In transfected LMH cells, all produced similar, if not higher (DuHe3, Du-He4), amounts of intracellular RC- and double-stranded linear (dsL) progeny DNA (Figure 2A), and of secreted, *bona fide* enveloped particles. This was demonstrated by the strongly (at least 20-fold) increased labeling of the particle-borne virus genome in the presence versus absence of detergent during endogenous polymerase reactions (Figure 2B). These reactions rely on the ability of the genome-linked (endogenous) P protein to incorporate exogeneously added dNTPs into the incomplete genome yet only if dNTP access to the nucleocapsid lumen is not blocked by an intact envelope.

### Heterologous replacement of the long, though not the short, form of the supposed HDR reduces infectivity of DHBV for cultured duck hepatocytes

**Figure 3:**
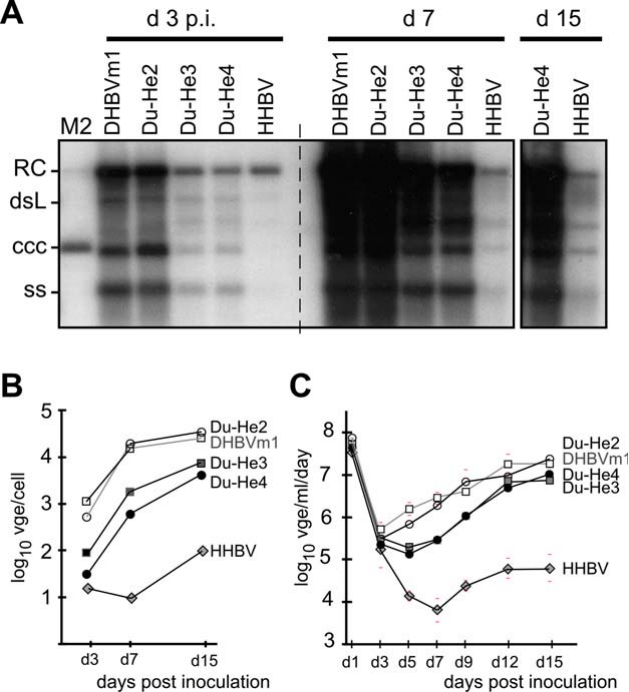
In vitro infectivity of chimeric viruses. PDH were incubated with the recombinant viruses at MOI 100. Cells were harvested at the indicated days post inoculation (p.i.). (A) Southern blot for intracellular viral DNAs. Abbreviations of the different DNA forms are as in the legend to Figure 2A. For day 15 p.i., only the lanes with Du-He4 and HHBV are shown. (B) Quantitation of intracellular viral DNAs by qPCR. Viral DNA amounts were normalized to cell numbers by qPCR as detailed in Protocol S1; for the early time points of the poorly infectious samples the values are maximum estimates due to the presence of some RC DNA from the inocula. (C) Quantitation of progeny virus production by qPCR. Viral titers are given as vge per ml culture fluid from approximately 10^6^ cells per day. Standard deviations (bars) are based on at least two, and usually four to five independent determinations.

Titers of transfection-derived virions were quantified as viral genome equivalents (vge); multiplicities of infection (MOI) were operationally defined as vge per inoculated cell. PDH were inoculated at MOI 100 with the recombinant chimeras, and recombinant DHBVm1 and HHBV as controls. Infectivity and the kinetics of infection (Figure 3) were scored by Southern blotting and by quantitative PCR (qPCR) [42] of intracellular viral DNAs, and of secreted vge between day 1 and day 15 post inoculation (p.i.); genotypes were confirmed by restriction analysis (Figure S1B).

Du-He2 behaved in all aspects indistinguishably from the reference virus DHBVm1. Already at day 3 p.i. both gave strong cccDNA and ssDNA signals; while the RC DNA signals could originate, in part, from the inocula they were clearly enhanced compared the other three samples. Signal intensities increased about 10-fold to day 7, and further to day 15. Both Du-He3 and Du-He4 produced 10- to 30-fold weaker signals until day 7 p.i.; thereafter the difference decreased to 4- to 6-fold, probably because DHBVm1 and Du-He2 had already reached a plateau. A similar around 10-fold reduction in PDH infectivity was observed for Du-He7 which had also not displayed any obvious defects in replication or envelopment in transfected LMH cells (data not shown). Signals for wt-HHBV remained two to three orders of magnitude below those from DHBVm1; however, infection was detectable by formation of small amounts of cccDNA and an about 10-fold net increase in intracellular and secreted viral DNAs from day 7 to day 15 p.i.. Thus, as expected if D-PreS 22–90 was important for the host tropism of DHBV, its exchange, or that of D-PreS 38–90 (Du-He3) or 91–161 (Du-He7), by He-PreS sequence markedly reduced infectivity for PDH whereas D-PreS 22–37 could be replaced without any negative impact; from this one would conclude that D-PreS 22–37 does not contribute to host discrimination. This was surprising given the reported stimulatory effect of D-PreS 22–37 on HHBV pseudotype infectivity for PDH and prompted us to repeat and extend the original pseudotyping experiments [23].

### Specific D-PreS segments including aa 22–37 can enhance PDH infectivity of HHBV pseudotypes

**Figure 4:**
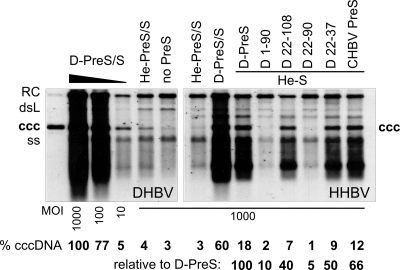
Selected D-PreS segments can enhance PDH infectivity of HHBV pseudotypes. PDH were inoculated with env^−^ DHBV (left panel) and HHBV genomes (right panel) pseudotyped with the indicated envelope proteins. Cells were harvested at day 7 p.i., and intracellular viral DNAs were analyzed by Southern blotting. He-S indicates that the L proteins consisted of an HHBV S domain combined with the indicated PreS domains; no PreS, complementation with DHBV S only. Levels of cccDNA were determined by phosphorimaging and are given relative to autologously pseudotyped DHBV at MOI 1,000 (upper lane), and to D-PreS/He-S pseudotyped HHBV.

Pseudotypes were generated by complementation of env^−^ HHBV and DHBV genomes with the envelope proteins of wild-type DHBV and HHBV, and with chimeric HHBV PreS/S proteins carrying D-Pres 1–90 and 22–108 [23], 22–37 and 22–90, or the PreS domain from crane HBV (CHBV1; Genbank accession: AJ441111) which is reportedly infectious for PDH [22]. Pseudotype yields were all similar, as determined by quantitation of envelope-protected viral DNAs. Autologously pseudotyped DHBV was applied at MOI 1000, 100 and 10 to calibrate PDH infection efficiency, all others were used at MOI 1000. Supernatant from cells transfected with the env^−^ DHBV genome plus a vector encoding only DHBV S (PreS^−^) served as background control. Cells were harvested at day 7 p.i. and analyzed for intracellular virus DNA by Southern blotting (Figure 4).

Though the RC-DNA signals were less informative (see above), the levels of the other viral DNA forms, including cccDNA, differed drastically, confirming in part the previously reported results. Autologously pseudotyped env^−^ DHBV gave still a clear cccDNA signal at MOI 10, slightly exceeding that obtained with HHBV PreS/S at MOI 1000; hence, as reported [23] the heron virus envelope conferred an about 100- to 1,000-fold reduced PDH infectivity. Infectivity of HHBV env^−^ was rescued most effectively by a complete DHBV envelope (D-PreS/S) yet, even in combination with HHBV S, the entire D-PreS domain and its segments 22–37, 22–108, as well as CHBV-PreS led to significant enhancements whereas D-PreS 1–90 and D-PreS 22–90 did not. These data were corroborated by qPCR of the intracellular replicative intermediates (RIs) although the accuracy of the low values was probably limited by contributions from inoculum derived RC-DNA. We cannot offer a trivial explanation for the discordant data regarding D-PreS 1–90; the sequence of the expression vector was correct, and enveloped particle formation was as efficient as with the other constructs. Most importantly, however, these data confirmed that D-PreS 22–108, and in particular D-PreS 22–37, increased infectivity of the HHBV envelope for PDH nearly as efficiently as the entire D-PreS domain. Thus in this test system introducing the D-PreS 22–37 segment into the HHBV envelope did affect host-specific infectivity whereas its reciprocal removal from the DHBV envelope in Du-He2 did not.

### Two chimeras with heterologous PreS sequences that cause poor PDH infectivity are highly infectious in vivo

To test in vivo infectivity of the chimeric viruses, three 3 day old ducklings each were inoculated with 10^8^ vge of recombinant chimeras Du-He2, Du-He3, and Du-He4; 7.5×10^7^ vge are estimated to be sufficient to deliver virus to about 10% of liver cells in 1-day-old ducklings [40]. Controls included recombinant wt-HHBV, DHBV16 and DHBVm1, and serum DHBV3 (Genbank accession: DQ195079) from a congenitally infected duck (10^9^ vge). Between one and three animals from each group, except those inoculated with HHBV, showed detectable surface protein antigenemia at day 7 p.i., and remained viremic until the end of the experiment at week 15; intrahepatic virus DNA levels at this time-point were comparable, regardless of the specific PreS sequence (data not shown). Genotypes were confirmed by restriction analysis, excluding wild-type virus as a source of infection in the animals inoculated with the chimeras (Figure S2A). None of the animals showed any overt pathogenicity (no major weight reduction or retarded growth [43], no gross abnormalities of the inner organs). Two persistently Du-He4 infected females were kept for more than one year, without significant decline in viremia. Hence even the poorly PDH infectious chimeras Du-He3 and Du-He4 were able to establish persistent infections in ducks; notably though, no signs of infection were detectable in 6 animals inoculated under identical conditions with Du-He7 (not shown).

### Efficient horizontal and vertical transmission of Du-He4 virus in ducks

**Figure 5:**
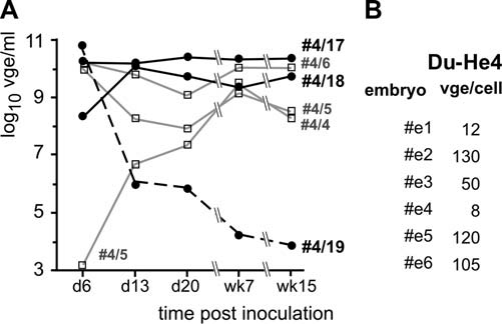
In vivo transmission of Du-He4 virus in ducks. (A) Horizontal transmission. Three ducklings each were inoculated with serum from a duck previously infected with recombinant Du-He4 virus (animals #4/17, #4/18, #4/19: 10^8^ vge; black lines), or with recombinant tagged wild-type DHBVm1 (animals #4/4, #4/6: 10^8^ vge; animal #4/5: 10^5^ vge; grey lines). Viremia was monitored over 15 weeks by qPCR. (B) Vertical transmission. Two persistently Du-He4 infected females were crossed with a DHBV-negative drake. Six off-spring embryos were sacrificed before hatch and their livers were analyzed for viral DNA. Du-He4 DNA loads were quantitated by qPCR and are given as vge/cell for each of the embryos.

To test whether Du-He4 could horizontally and vertically be transmitted, three ducklings (#4/17 to #4/19) were inoculated with Du-He4 positive serum from one of the above described animals (10^8^ vge per animal); serum containing DHBVm1 served as control (animals #4/4 and #4/6 received 10^8^ vge, animal #4/5 10^5^ vge). Viremia (Figure 5A) was already high at day 6 p.i. in all animals inoculated with 10^8^ vge; viral load in animal #4/5 was initially low but increased to similar levels by week 7. Du-He4 viremia in animal #4/19 declined, yet the two others maintained very high viral loads (around 10^10^ vge/ml) over 15 weeks.

For vertical transmission two persistently Du-He4 infected females were crossed with a DHBV negative drake. Six off-spring embryos were sacrificed 5 to 9 days before hatch (d 28), and viral DNA in the livers was analyzed for Du-He4 genotype (Figure S2B) and quantitated by qPCR. All samples were positive for Du-He4 DNA (Figure 5B). Hence chimera Du-He4 could horizontally and vertically be transmitted in ducks.

### Chimeric envelope of Du-He4 mediates poor infectivity for PDH but allows efficient spread in ducks

**Figure 6:**
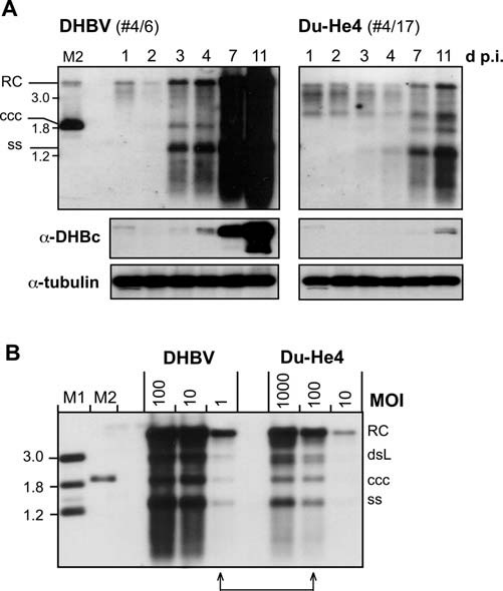
Poor in vitro infectivity of Du-He4 is not an artifact of the recombinant virions. PDH were inoculated with serum-derived rather than recombinant virions (DHBVm1 from animal #4/6; Du-He4 from animal #4/17). (A) Kinetics of infection. PDH inoculated with DHBV or Du-He4 at MOI 100 were harvested at the indicated time points. Viral DNAs were analyzed by Southern blotting. The panels below show Western blot signals for DHBV core protein (using mAb 2B9 4F8 [71]) and tubulin from the same lysates used to prepare viral DNA. (B) Du-He4 envelope is at least ten-fold less efficient than the wild-type DHBV envelope in mediating PDH infection. PDH were incubated with the two viruses at the indicated MOIs. Cells were harvested 7 days p.i., and viral DNAs were analyzed by Southern blotting. Note that signals for Du-He4 at MOI 100 were only slightly stronger than for DHBV at MOI 1 (connected arrows).

**Figure 7:**
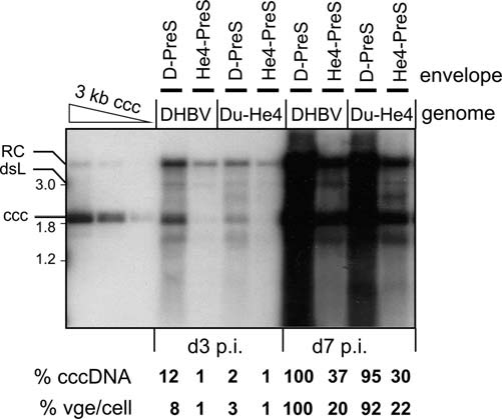
Poor in vitro infectivity of Du-He4 is largely caused by the chimeric envelope. The env^−^ DHBV genome and a correspondingly mutated Du-He4 genome were trans-complemented by the authentic D-PreS/S envelope or the chimeric Du-He4-PreS/S envelope, and the resulting pseudotypes were used to inoculate PDH at MOI 100. Cells were harvested at day 3 or day 7 p.i., and viral DNAs were analyzed by Southern blotting and quantitation by phosphorimaging of the cccDNA signals, assigned by a dilution series of a 3 kb DHBV sequence containing plasmid DNA and by qPCR of intracellular RIs. The corresponding values, normalized to those obtained with the D-PreS/S complemented DHBV genome at day 7 p.i. set at 100%, are given below each lane. Note that low signal intensities resulted largely from the chimeric Du-He4 envelope, not the chimeric genome.

The disparate in vitro versus in vivo results for Du-He4 prompted us to thoroughly exclude that we had underestimated its in vitro or overestimated its in vivo infectivity. Improper modification of the chicken LMH cell-derived Du-He4 virions did not account for poor PDH infectivity because Du-He4 virions sequentially passaged through two ducks were similarly attenuated (Figure 6A and 6B); this makes it also unlikely that the fast in vivo spread of Du-He4 (see below) resulted from quickly arising adaptive mutations not detectable by restriction genotyping. A significant replication defect of the Du-He4 genome in PDH that went unnoticed in LMH cells was ruled out by pseudotyping the env^−^ DHBV genome, and an analogous env^−^ Du-He4 genome, with either wild-type D-PreS/S, or with Du-He4 PreS/S. The chimeric envelope was up to ten-fold less efficient in rescueing infectivity of both env^−^ genomes whereas replication of the Du-He4 genome was only slightly retarded at day 3 p.i. but reached DHBV-like levels at day 7 p.i. in both settings (Figure 7).

**Figure 8:**
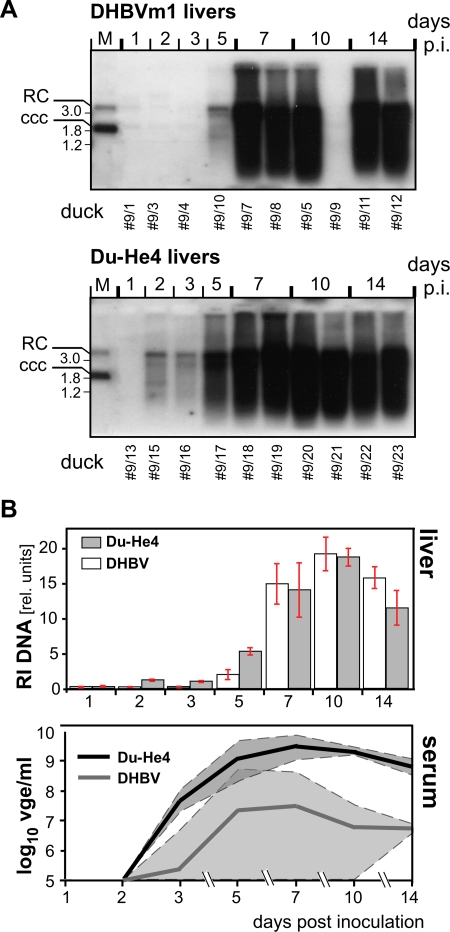
Quantitative comparison of in vivo spread of Du-He4 versus wild-type DHBV. Ten ducklings each were inoculated with serum containing 10^8^ vge of Du-He4 (from animal #4/17) or DHBVm1 (from animal #4/6). One animal each was sacrificed at days 1, 2, 3, and 5 p.i. and two animals each at days 7, 10, and 14 p.i.; intrahepatic virus DNA and viremia were quantitatively monitored by Southern blot and DNA dot blot, respectively. Animal #9/9 from the DHBVm1 group showed no signs of productive infection and was excluded from further analysis. (A) Intrahepatic DNA. DNA from liver autopsies was analyzed by Southern blotting. (B) Quantitation of intrahepatic and circulating virus DNA. Signal intensities from appropriate exposures of the Southern blots shown in A were quantitated by phosphorimaging and normalized to an identical amount of DHBV marker DNA present on both gels. Error bars indicate the deviation between two independent DNA preparations from one animal (samples from days 1, 2, 3, 5, and day 10 for DHBVm1) or from two independent DNA preparations from two animals (all others). To monitor viremia, serum samples were collected at all indicated time points until the respective animal was sacrificed. Titers were determined by DNA dot blot (Figure S3) and phosphorimaging. The thick lines represent the mean values of all samples from one group, the dashed lines the minimal and maximal values from individual samples.

In the previous in vivo experiments, Du-He4 had reached wt-DHBV-like viral loads (Figure 4A) but slowed-down kinetics of spread would not have been detected. We therefore monitored the spread of both viruses, in liver and serum, at shortly spaced intervals. Ten ducklings each received 10^8^ vge of serum-derived Du-He4 (from duck #4/17) or DHBVm1 (from duck #4/6). One animal each was sacrificed at days 1, 2, 3 and 5 p.i., and two animals each at days 7, 10 and 14 p.i.. Intrahepatic viral DNA was analyzed by Southern blotting (Figure 8A), and the course of viremia by DNA dot blot of serum samples (Figure S3); both data sets were quantitated by phosphorimaging (Figure 8B). For DHBVm1, intrahepatic virus DNA became clearly detectable at day 5, then rapidly rose to maximal values at day 10, and slightly declined. Du-He4 produced a very similar profile, except that signals at the early timepoints (days 2 to 5; see Figure 8A) were even stronger. Viremia was monitored in individual serum samples from all animals alive at a given time-point (i.e. ten for day 1, two for day 14). The mean and maximal values were again higher and displayed a faster rise for the chimera than for the wild-type virus. While the viremia values are statistically significant, especially for the early time points covering samples form several ducks, each liver data is derived from an individual duck and thus should not be overinterpreted to indicate superiority of one virus over the other. However, if the about 10-fold lower PDH infectivity of Du-He4 had translated into a 10-fold lower percentage of initially infected cells, i.e. about 1% instead of 10% [40], the appearance of detectable levels of the chimeric virus should have occurred with a substantial delay. For wild-type DHBV in young ducklings, the mean doubling time in the percentage of infected hepatocytes has been estimated to be about 16 h [40], thus three to four times this time (2 to 3 d) would have been required for Du-He4 to catch up with the wild-type virus, provided replication *per se* proceeded at similar rates. As no such delay was observed, wild-type and chimeric virus appear to spread with comparable kinetics.

In these experiments the viral loads showed less variation with Du-He4 than with wild-type DHBV where such variability has been seen also in other studies [44]. Because DHBV is wide-spread in commercial flocks, including the animals in the current study, mother-to-egg transmission of anti-DHBV antibodies, usually directed against the envelope [29], could reduce or prevent viral spread in some of the ducklings [30]. To test whether Du-He4 represented an *a priori* immune escape variant we preincubated Du-He4, or DHBVm1 containing sera with a previously characterized DHBV neutralizing duck antiserum obtained by DNA immunization with a DHBV16 PreS/S expression vector and shown to react predominantly with PreS rather than S [28], or for control with normal duck serum. Aliquots containing 10^6^ vge Du-He4 were inoculated into two ducklings each. In the test animals (#7/1, #7/2), viral loads as determined by qPCR (Figure S4) remained close to or at the detection limit of our non-nested qPCR assay (about 100 vge per PCR reaction, corresponding to 10^5^ vge/ml per 1 µl serum sample) between day 4 and day 11 p.i.; by day 28, serum from one animal was essentially negative for viral DNA, the other had developed a low titered viremia of 1.6×10^5^ vge/ml. In contrast, both control animals (#7/3, #7/4) developed DHBs antigenemia, easily detectable by day 11 and day 28 p.i., and viremia which peaked at 2.2 and 6.5×10^8^ vge/ml on day 11. The antiserum also strongly reduced infectivity in one (#7/6), and partially in the second (#7/5) DHBVm1 inoculated duck, whereas the control animals (#7/7,8) developed antigenemia and viremia at a similar rate and to similar levels as the Du-He4 controls (Figure S4). These data suggest that antibodies raised against the wild-type DHBV surface proteins can also neutralize the chimeric virus; in addition, they demonstrated that 10^6^ vge of Du-He4 could establish in vivo infection in ducks.

### Estimating the specific infectivity of the Du-He4 chimera

One wild-type DHBV genome has been proposed to be infectious in neonatal ducks [40]; for the human virus, the doses required to infect 50% of the inoculated chimpanzees (CID_50_) have been reported to range between 3 and 169 vge [45]. It was therefore of interest to see whether replacement of the supposed HDR in Du-He4, though principally compatible with in vivo infectivity (see above), negatively affected specific infectivity. To this end, 6 ducklings each were inoculated with DHBVm1 (from duck #2/7) and Du-He4 (from duck #2/20) viremic sera, diluted with normal duck serum to contain 10^7^, 10^5.5^, 10^4^, and 10^2.5^ ( = 300) vge per dose. Development of antigenemia and viremia was monitored for up to 42 days p.i.; at the end of the experiment, animals were sacrificed and the livers were removed for determination of intrahepatic viral DNA.

**Figure 9:**
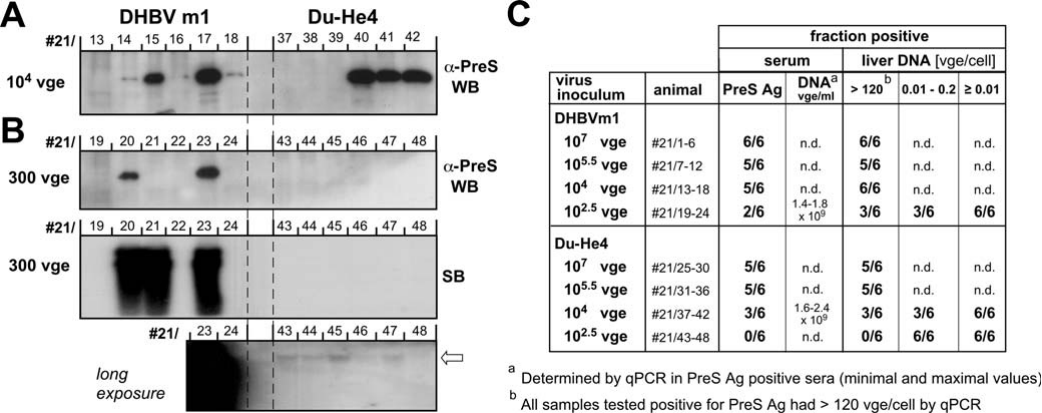
Estimating the specific in vivo infectivity of Du-He4 versus wild-type DHBV. (A) PreS antigenemia after inoculation with 10^4^ vge of DHBVm1 or Du-He4. PreS antigenemia was monitored by Western blotting with mAb 4F8, using 0.5 µl of the final sera from the indicated animals per lane; birds #21/14, 16, and 18 were scored positive because the signals, though weak, were above background. (B) Antigenemia and intrahepatic viral DNA after inoculation with 300 vge of DHBVm1 or Du-He4. PreS/S antigenemia was monitored as in A. Intrahepatic DNA was analyzed by Southern blotting, using 5 µg of total liver DNA per sample. The long exposure (bottom panel; exposed 6 times longer and using an intensifying screen) revealed weak signals at the expected position for all ducks inoculated with 300 vge of Du-He4 (arrow); a corresponding signal for duck #21/24 from the wild-type group is covered by the strong signal from the neighboring #21/23 sample. The presence of viral DNA in all samples was confirmed by qPCR. (C) Summary of fraction of birds positive for individual infection markers. Numbers from the column PreS Ag were used to calculate formal ID_50_ values.

Analysis of the serum samples revealed that at the highest dose, 6 of 6 animals from the DHBVm1 and 5 of 6 animals from the Du-He4 group had developed antigenemia and viremia, easily detectable by day 12 p.i. and persisting to end of the experiment; for the 10^5.5^ inocula, 5 of 6 animals in each group were positive for serum markers of infection. Slight differences became apparent at the lowest doses (Figure 9A); with the 10^4^ vge inocula 3 of 6 Du-He4 inoculated versus 5 of 6 DHBVm1 inoculated birds developed antigenemia and viremia (titers in all cases around or slightly above 10^9^ vge/ml); with the 300 vge inocula, numbers were none out of 6 versus 2 out of 6. Formal ID_50_ values as calculated from the fraction of animals positive for serum PreS antigen at the end of the experiment by the Spearman-Kärber method [46] were 10^3.3^ (about 2,000; 95% confidence interval 10^2.2^–10^3.8^) vge for the wild-type virus and 10^4.5^ (about 30,000; 95% confidence interval 10^3,6^–10^5,4^) vge for Du-He4; the Reed-Münch method [47] yielded ID_50_ values of 10^3^ for wild-type DHBV and 10^4^ vge for Du-He4. Though only a trend given the limited number of animals, this was compatible with a somewhat reduced efficiency of Du-He4 in establishing an overt in vivo infection at low inoculum doses.

However, in their livers all animals inoculated with 300 vge of Du-He4, as well the wild-type DHBV inoculated ducks that had remained negative for serum markers of infection, contained very low but Southern blot detectable amounts of viral DNA (Figure 9A, bottom panel). As determined by qPCR, the livers of the serum antigen positive ducks contained at least 120, and usually around 200 vge/cell, as did the liver of animal #21/21 from the wild-type virus group that was negative for serum antigen but had a low level viremia (7×10^6^ vge/ml versus about 1.5×10^9^ vge/ml for the antigenemic ducks). Livers from all other animals contained between 0.01 and 0.20 vge/cell. Though the accuracy of these numbers is limited, even the lowest values were at least five times above background. Most importantly, extrapolated to the entire liver (liver weights 30–50 g at the time the animals were sacrificed) and assuming 7×10^8^ cells per gram liver [48],[49], even 1 vge per 100 cells translates into a total of 2×10^8^ vge per animal, indicating a 6 log amplification of the input virus. Because according to these data all 6 ducklings receiving 300 vge of Du-He4 were initially infected, as were the four non-antigenemic wild-type DHBV inoculated ducks, a formal ID_50_ cannot be calculated; however, it must be well below 300 vge. Thus although a low dose infection with Du-He4 may be more effectively contained by the host than infection with wild-type DHBV, heterologous replacement of the PreS region supposedly critical for the duck tropism of DHBV had no major negative impact on specific infectivity in ducks.

## Discussion

The essential role of the preS domains for hepadnavirus infection is indisputable. Hence the model that a specific segment within PreS determines host range is plausible, and it is experimentally supported by pseudotype - PDH infection data [23]. Our results confirm significant aspects of these previous findings, including the ability of D-PreS 22–37 to enhance PDH infectivity of heterologous pseudotypes and thus to affect host specific infectivity in that test system; however, they also preclude the generalized conclusion that this sequence, or its longer counterpart 22–90, constitutes the avihepdnaviral host-determining region. In the context of the DHBV envelope, replacement of D-PreS 22–37 by HHBV sequence (Du-He2) had no negative impact on infectivity at all and, most importantly, a duck virus chimera in which the entire region suggested to determine duck tropism was replaced by heron virus sequence (Du-He4) was highly infectious for ducks. Hence the factors that determine infectivity in vitro are not the same as in vivo. Our study therefore unveils fundamental gaps in the current understanding of hepadnaviral host tropism.

### Individual PreS segments affect infectivity for isolated hepatocytes in a non-reciprocal and non-linear fashion

An absolute requirement for the infection experiments was that the chimeras be able to produce intact enveloped virions. None of the chimeras reported here displayed any obvious defects, as most compellingly demonstrated by their in vivo infectivity.

Nonetheless, chimeras Du-He3 and Du-He4, but not Du-He2, were poorly infectious for PDH; these in vitro data, performed on the same batch of PDH for the different viruses and reproduced several times, are statistically highly significant (P<0.001, Student's t-test). Hence replacement of D-PreS 22–90, 38–90, and also 91–160 (Du-He7; not shown), by He-PreS segments substantially reduced infectivity for PDH but replacement of D-PreS 22–37 did not. Simply interpreted, DHBV specific sequences downstream of aa 37 were required for efficient infection of duck hepatocytes but the sequence 22–37 was not. Thus in this test system, D-PreS 22–37 had no species-specific impact although the same sequence significantly enhanced PDH infectivity of the HHBV pseudotypes; in other words, reciprocal exchanges of PreS segment 22–37 did not produce reciprocal phenotypes.

Other data sets also elude a simple interpretation. D-PreS 22–37 and 22–108 enhanced PDH infectivity of HHBV pseudotypes as reported [23], yet 22–90, and 1–90, did not; pseudotype formation with these latter constructs was as efficient as with the others, and numerous of the pseudotypes prepared and tested in parallel (Figure 4) clearly scored positive. Thus there is no linear relationship between the proportion of a given PreS sequence and infectivity for hepatocytes from the homologous host. This suggests that consecutive PreS segments act as multimodular three-dimensional, not linear, structures that may undergo complex and dynamic intra- and intermolecular interactions. Given the limited [50] or absent knowledge on the structure of PreS and its relevant cellular partners, attempts to define a linear sequence segment as critical for host range determination therefore appear to be doomed.

This conclusion is supported by in vitro infectivity data obtained with hepatitis delta virus particles carrying chimeric hepadnavirus envelopes [51]. Replacement of the N terminal HBV PreS1 segment 1–40 by that from WMHBV reduced, indeed, HDV infectivity for human hepatocytes, in accord with a previous HBV pseudotype study [36]; however, infectivity for spider monkey cells was likewise reduced, although in vivo WMHBV can infect the woolly monkey related spider monkeys but not chimps [52]. Even more surprisingly, reciprocal introduction of human virus PreS1 1–40 into a WMHBV envelope did not enhance infectivity for human cells yet even strongly boosted infectivity for spider monkey hepatocytes [37]. These data cannot be reconciled with the simple concept of a defined sequence segment in PreS acting as a host range determinant.

The most compelling counterevidence, however, comes from the in vivo infection experiments in this study. In ducks, Du-He4 established high-titered persistent infections; the kinetics of intrahepatic spread after inoculation with 10^8^ vge/duckling were comparable and, in no case, slower than with wild-type DHBV (Figure 8). At inoculum doses of 10,000 or 300 vge/animal, wild-type DHBV seemed to be somewhat more efficient regarding the fractions of birds developping antigenemia and viremia (Figure 9), with formal ID_50_ values of around 10^4^ for Du-He4 vs. 10^3^ for wild-type DHBV; however, the small number of animals imposes the same limitation on such an interpretation as the seemingly better performance of the chimera at the 10^8^ vge inocula. Most importantly, all animals inoculated with only 300 vge and negative for serum markers of infection contained intrahepatic viral DNA. Because the absolute copy numbers determined by qPCR were low (350 to 2,000 copies per PCR reaction) the estimates of between 0.2 and 0.01 vge per cell may not be very accurate. Clearly, however, even one vge per 100 cells amounts to a total of more than 10^8^ vge per duck liver [49], demonstrating a 10^6^-fold, or higher, amplification of the inoculum. Thus 300 vge of Du-He4, as well as of wild-type DHBV, were sufficient for an at least initial infection of 6 out of 6 ducklings. This prevented calculation of a formal ID_50_ value; as an approximation to the upper limit of the actual ID_50_ it may be assumed that the next lower dilution would have infected only a fraction of animals; in our experiments, this would correspond to an input of 10 vge per animal, close to the value reported for wild-type DHBV [40].

Whether or not the chimeric genomes accumulated mutations, and if so which, upon multiple passages in the duck is currently being investigated using serial samples, yet maintainance of the low PDH infectivity phenotype after passage in ducks (Figure 6) as well as preliminary sequencing data make it unlikely that rapidly occurring adaptive mutations [53] caused the high in vivo infectivity. Thus, Du-He4 is a fully functional duck-infectious avihepadnavirus although nearly half its PreS sequence is from a virus that does not infect ducks and although the heterologous sequence clearly reduced infectivity for duck hepatocytes. Thus the predictive significance of in vitro infectivity data for in vivo infectivity is strongly limited.

### Implications of distinct in vitro versus in vivo infectivity of chimera Du-He4

The high in vivo versus low in vitro infectivity of Du-He4 points to major differences between the two settings even though the primary hepatocytes originated from the same species as the experimental animals. In fact, even for wt-DHBV the amounts of virus required for PDH infection appear high given that few DHB virions can establish infection in ducks ([40] and this study), and this difference seems to be further magnified in the Du-He3 and Du-He4 chimeras. Possibly, the expression profiles of cell factors required for, or restricting infection [5], such as those controlling retroviruses [54],[55], differ between hepatocytes in culture versus in the liver, and the chimeras are more sensitive to such alterations. Alternatively, the intact liver may have a higher capacity for as yet undefined processing steps in the envelope that increase infectivity, or its tissue architecture provides for particularly intimate contacts between hepatocytes and incoming virus that outbalance a reduced affinity of the chimeric preS regions for the unknown receptors; it has even been proposed that liver sinusoidal endothelial cells, not hepatocytes, are the initial in vivo target cells [56], yet the physiological relevance of this model is unclear. For several viruses, spread is particularly efficient via direct cell-to cell contacts, such as filopodial bridges [57] or the recently described nanotubes [58]; such processes may also contribute to the efficiency of hepadnaviral spread in vivo yet not, or less so, in cultured hepatocytes.

Another conceivable option for the high in vivo infectivity of Du-He4 was that the chimera had a selective advantage relating to preexisting (possibly present in some of our animals), or rapidly inducible antibodies that neutralize the wild-type virus [31] but not the chimera. Our neutralization experiments argue against but do not fully exclude this possibility. The antiserum used reacted on Western blots virtually exclusively with the L protein but not the S protein of DHBV, as reported [28], and the chimeric Du-He4 L protein was recognized with similar efficiency. Though in accord with its neutralizing activity against Du-He4, it is not clear whether the fraction of Western blot reactive antibodies in the antiserum, or possibly others directed against the native L and/or S protein, are the relevant ones for neutralization, and the fraction of these relevant antibodies might differ for wild-type DHBV and Du-He4.

Notably, Du-He3 and Du-He4 infectivity for PDH was not as strongly reduced (by around 10-fold) as that of HHBV (>100-fold), which might suggest that there is a threshold in vitro infectivity that is compatible with in vivo infection. Along this line, approximate quantitation of the PDH infectivities of recombinant Ross' Goose (RGHBV; Genbank accession: AY494848), snow goose (SGHBV; Genbank accession: AF110997), and stork hepatitis B (STHBV; Genbank accession: AJ251937) viruses showed reductions of 8 to 20-fold for the goose viruses and >100-fold for STHBV (K. Dallmeier and M. Nassal, unpublished data). While, to our knowledge, no in vivo infection data have been published for these viruses, a virus almost identical to RGHBV isolated from Mandarin ducks was indeed infectious for ducks [59], and STHBV would be predicted not to infect ducks. Thus an about 10-fold reduction in the efficiency of virus uptake, which is probably what is reflected by the data obtained on isolated hepatocytes, may not be rate-limiting for virus spread in vivo.

However, not any virus displaying an intermediate (as opposed to a drastic) reduction in in vitro infectivity is able to establish in vivo infection. Of 6 ducklings inoculated with 10^8^ vge of chimera Du-He7 none developed signs of infection although the chimera produced similar amounts of enveloped particles in transfected LMH cells and had a similar in vitro infectivity as Du-He3 and Du-He4. The same was observed with a DHBV chimera in which the core gene was replaced by that from HHBV (Dallmeier and Nassal, unpublished). Similarly, a natural avihepadnavirus isolate from an Ashy-headed sheldgoose (ASHBV; Genbank accession: NC_005890) with reduced but clearly detectable infectivity for PDH [59] failed to infect 6 out of 6 ducklings. Hence there seems to be no simple correlation between in vitro and in vivo infectivity. Given these various uncertainties, testing in vivo infectivity appears currently as the only reliable way of addressing hepadnaviral host range.

This conclusion may be relevant for the risk assessment of vaccine escape and drug resistant variants of human HBV. De novo infection with such mutants could seriously impair efficacy of vaccination and restrict the options for chronic hepatitis B treatment [60]. Some resistance-conferring mutations seem to lower replication capacity [61], yet their effect on infectivity is not known. Notably, for HBV the discrepancy between highly efficient in vivo infection [45] and the requirement for MOIs of 10 or higher, and often for polyethylenglykol as an additional facilitator [51],[62],[63], in the current cell culture infection systems is even more pronounced than for DHBV. Hence cell culture data may not be dependably extrapolated to the behaviour of such viruses in vivo. Even if our in vivo titration data were interpreted as evidence for a somewhat reduced specific infectivity of Du-He4, this was manifest only at inoculum doses of 10^4^ vge or less; given that HBV titers in humans frequently exceed 10^9^ vge per ml, such a dose would be contained within 10 nl of blood, only a fraction of what is commonly transferred in needlestick injuries with even the finest (25G) needles [64]. Hence fundamental questions in hepadnavirus infection biology will need to be reassessed, not only regarding the identity of the still obscure cellular factors involved but also with respect to the seemingly well understood role of the viral envelope proteins in mediating infectivity and tissue and host tropism.

## Materials and Methods

Full protocols and details of plasmid constructions are supplied as Protocol S1 in Supporting Information.

### Expression constructs for recombinant viruses and envelope proteins

Virus expression vectors were based on plasmid pCD16, carrying a CMV promoter controlled 1.1× DHBV16 genome [65]; the tagged derivative DHBVm1 contained three nt substitutions that created new restriction sites for BsmBI and MroI (Figure S1). Chimeric DHBV genomes encoded the following aa replacements: Du-He2, D-PreS 22–37 by He-PreS 22–40; Du-He3, D-PreS 38–90 by He-PreS 38–92; Du-He4: D-PreS 22–90 by He-PreS 22–92 (Figure 1C). Vector pCHHBV4 was based on HHBV4 (Genbank accession: NC_001486) [24]. Envelope-deficient (env^−^) DHBV and HHBV genomes corresponded to those described earlier [23],[33],[66]. PreS/S expression vectors were all controlled by the CMV promoter; the CHBV PreS construct contained a segment from CHBV1 [22], kindly provided by H. Sirma and H. Will.

### Replication competence and enveloped virion formation

Intracellular replicative intermediates (RIs) from transfected LMH cells were analyzed by Southern blotting of DNAs from cytoplasmic extracts; secreted virions were enriched by D-PreS-specific immunoprecipitation, then subjected to endogenous polymerase assay conditions [67],[68] with or without detergent. Alternatively, viral DNA in enveloped particles was enriched by pronase plus nuclease treatment [33] and used for quantitative PCR (qPCR) determinations. Recombinant viruses were obtained analogously; pseudotypes were generated by cotransfection of vectors encoding an env^−^ viral genome and the desired PreS/S proteins. Viral titers, as viral genome equivalents per ml (vge/ml), were determined by DNA dot blot or by qPCR.

### In vitro infections

Primary duck hepatocytes [69] were usually incubated overnight at the desired MOI with recombinant virus, or with viremic duck serum. For analysis of intracellular viral DNAs including cccDNA, total DNA was prepared by SDS lysis [66].

### In vivo infections

Two to three day old Pekin ducklings were injected into the foot vein with the indicated amounts of recombinant virus or viremic serum. Viremia was determined as described above for cell culture supernatants. To monitor the kinetics of viral spread in the liver, one or two animals each were sacrificed at each time-point, and liver DNA was analyzed by virus specific Southern blot. To estimate specific infectivities, six ducklings each were inoculated with viremic sera diluted to contain 10^7^, 10^5.5^, 10^4^, and 10^2.5^ vge per dose of either the reference virus DHBVm1 or Du-He4. Serum samples were collected for up to 42 days, and PreS antigemia was monitored by Western blotting; viral loads in selected PreS antigen positive animals were determined by qPCR. At the end of the experiment, the animals were killed and their livers were removed to monitor intrahepatic viral DNA by Southern blotting and by qPCR. The fractions of animals positive for serum PreS antigen were used to calculate formal ID_50_ values using the Spearman-Kärber [46] and the Reed-Muench method [47]. All animal experiments were approved by the local authorities (Regierungspräsidium Freiburg, project G02/36) and performed in compliance with German animal welfare legislation at a registered facility of the University Hospital Freiburg under veterinary supervision.

## Supporting Information

Protocol S1 Supporting Materials and Methods. This file contains a detailed description of experimental procedures not contained in the brief general M+M section of the main text, plus a list of supporting references.
(0.14 MB DOC)

Figure S1 Genetic tags for discrimination of chimeric from wild-type viruses. A. Partial restriction maps of preS-derived PCR amplicons from the viruses used in this study. DHBV16 and DHBV3 are the predominant wild-type DHBV strains endemic in the US and Europe, respectively. The tagged DHBV16 derivative DHBVm1, used as reference wild-type virus, contains the artificially introduced Bsm BI site present in all chimeras, and the Mro I site present in Du-He3 and Du-He4. B. Genotyping of selected chimeras after in vitro infection of PDH. The example shows the restriction patterns of PCR amplicons obtained from intracellular nucleocapsid-associated DNA of PDH inoculated with the indicated recombinant viruses. Each amplicon produced the expected unique pattern. The same type of analysis was used to confirm infection by the desired virus variants in vivo and to exclude congenital infection by DHBV3 or contamination with the laboratory strain DHBV16.
(0.64 MB PDF)

Figure S2 Genetic tags are preserved upon in vivo infection of ducks with chimeras Du-He2, Du-He3 and Du-He4. A. Serum samples (day 7 p.i.) from ducks inoculated with the indicated recombinant viruses. PCR amplicons were incubated with the indicated restriction enzymes and the products were analyzed by agarose gel electrophoresis. B. Serum sample from vertical transmission experiment (embryo #e1). All samples produced input-virus specific fragment patterns (see Figure S1).
(0.99 MB PDF)

Figure S3 In vivo spread of Du-He4 is as fast as, or faster than that of wild-type DHBV. Ten ducklings each were inoculated with serum-derived DHBVm1 (from animal #4/6) or Du-He4 (from animal #4/17) and analyzed as described in the legend to Figure 8. Animal #9/9 from the DHBVm1 group showed no signs of productive infection and was excluded from further analyis. A. Kinetics of viremia. Serum samples from the indicated animals were collected at the indicated days p.i. and analyzed by DNA dot blot using a 32P labeled bispecific DNA probe; one out of several exposures is shown. The day 2 sample from animal #9/18 could not be analyzed. B. DNA dot blot of DHBV plasmid DNA standard used for calibration. A dilution series of plasmid pCD16 DNA containing the indicated amounts of viral genome equivalents was dotted on a membrane and detected with the identical probe as the dot blots shown in A. A graphic representation of the resulting values is shown in Figure 8B.
(0.41 MB PDF)

Figure S4 Du-He4 is neutralized by duck sera against wild-type DHBV L protein. Serum samples containing 107 vge/ml of Du-He4 (from animal #4/17) or DHBVm1 (from animal #4/6) were incubated with an equal volume of a previously characterized DHBV neutralizing duck antiserum (α-DPreS/S), or with normal duck serum (NDS), for 1 h at room temperature. Aliquots containing 106 vge were inoculated into two ducklings each (animals #7/1,2: Du-He4+α-DPreS/S; #7/3,4: Du-He4+NDS; #7/5,6: DHBVm1+α-DPreS/S; #7/7,8: DHBVm1+NDS). Viremia was monitored by qPCR for 28 d p.i.; values below 104 vge/ml are close to the detection limit and may not be very accurate. In animal #7/5 (DHBVm1+α-DPreS/S; not shown) development of viremia was delayed but an increase in viral load from 8.3×103 vge/ml at d 7 p.i. to 6.1×106 vge/ml at d 11 indicated partial protection.
(0.06 MB PDF)
