# Supplementary material for: Heterologous Replacement of the Supposed Host Determining Region of Avihepadnaviruses: High In Vivo Infectivity Despite Low Infectivity for Hepatocytes
Source: PLoS Pathog. 2008 Dec 5;4(12):e1000230. doi: 10.1371/journal.ppat.1000230 (PMC2585059; doi:10.1371/journal.ppat.1000230)
Supplement: Protocol S1 — Supporting Materials and Methods. This file contains a detailed description of experimental procedures not contained in the brief general M+M section of the main text, plus a list of supporting references. (0.14 MB DOC) [file ppat.1000230.s001.doc]

**PROTOCOL S1. Supporting Materials and Methods**

**Expression constructs for recombinant viruses and envelope proteins**

All virus expression vectors were based on plasmid pCD16, containing a 1.1x DHBV16 [1] genome under control of the CMV IE enhancer/promoter [2]; in DHBV env^-^, the TGG PreS codon for W122 was changed to TGA [3]. The HHBV vector pCHHBV4 contained a 1.2x HHBV4 [4] genome derived from plasmid 413-2 [5], kindly provided by D. Loeb. Derivative pCHHBV4n1 contained an additional Bst EII site at position 733, immediately upstream of the preS/S ORF, that is silent in *P*. In this vector, the TTC codon for F5 in S of pCHHBV4n1 was converted to TAA [6] to create HHBV env^-^. For the chimeric DHBV genomes, appropriate PCR fragments obtained on pCHHBV4 as template were cloned into pCD16 to create the following replacements: Du-He2, D-PreS 22-37 by He-PreS 22-40; Du-He3, D-PreS 38-90 by He-PreS 38-92; Du-He4: D-PreS 22-90 by He-PreS 22-92. PreS/S expression vectors were generated by transferring appropriate fragments from the virus vectors into plasmid pCDNA3.1+ (Invitrogen), or by deleting the region upstream of the preS/S gene (nt 2534 to 733) from the respective chimeric HHBV expression plasmids. Wild-type DHBV S and HHBV S expression vectors were obtained by deleting the preS ORFs. The CHBV construct was based on CHBV1 [7]. Further details of the constructions are available from the authors upon request.

**Genetically tagged virus genomes**

A summary of the restriction sites introduced into the various constructs is shown in Figure S1. The wild-type DHBV reference virus DHBVm1 contained a new Bsm BI site at nt 833 instead of the natural Aat II site at nt 828 in DHBV16, and an additional Mro I site at nt 909. The nt exchanges were silent in PreS/S and resulted in the amino acid substitutions Q222E and Q249K in the spacer region of P, respectively. A new Ngo MIV site at HHBV nt 864 served as marker for He-PreS 22-40 (chimeras Du-He2 and Du-He4), and the genuine HHBV Sac II site as a marker for He-PreS 38-92 (chimeras Du-He3 and Du-He4).

**Determination of replication competence and enveloped virion formation**

Most procedures used have previously been described [8,9]. In brief, LMH cells were transfected with the appropriate plasmids using FuGene6 reagent (Roche). Three to four days post transfection, cytoplasmic cell extracts were prepared using NP-40 lysis buffer (50 mM Tris-HCl [pH 7.5], 100 mM NaCl, 20 mM EDTA and 0.5% (v/v) Nonidet NP-40). Nucleocapsid-associated viral DNAs were prepared by overnight digestion of plasmid DNA with 0.5 mg/ml DNase I in the presence of 6 mM Mg^2+^ acetate, and analyzed by Southern blotting using a 1:1 mixture of ^32^P labeled DHBV and HHBV DNA as a bispecific probe, and by quantitative PCR (qPCR; see below). To account for potential differences in transfection efficiency, the amounts of viral DNA as determined by qPCR were normalized to the amounts of cytoplasmic core protein as detected by immunoblotting using the DHBV core protein specific monoclonal antibody (mAb) 2B9-4F8 [10], or a polyclonal rabbit antiserum that detects both DHBV and HHBV core proteins. No significant variation in the ratios of DNA to core protein was observed between the different constructs, indicating the chimeric P proteins and genomes resulting from PreS domain swapping were fully functional.

The presence of secreted enveloped virions was addressed by detergent-dependent dNTP incorporation in endogenous polymerase assays. Culture supernatants, collected from day 3 to day 5 post transfection, were subjected to immunoprecipitation with the D-PreS specific mAb 4F8 ([11], kindly provided by C. Kuhn and H. Schaller); except for wild-type HHBV, precipitation efficiencies were similar for all constructs, as determined by qPCR quantitation of the precipitated viral genomes. Then equal aliquots of the immunopellets were subjected to endogenous polymerase assay conditions [9,12] in the presence of α^32^P-dATP, either with, or without 0.5% (v/v) NP-40 detergent. Products were analyzed by agarose gel electrophoresis and quantitation by phosphorimaging (Fuji BAS 1500). Alternatively, an enzymatic procedure was used that enriches for viral DNA in enveloped particles [13] by removal of viral genomes in naked nucleocapsids and of plasmid DNA by combined incubation with DNase I plus 0.5 mg/ml of Pronase E. This material was also used for as template for qPCR.

**Recombinant viruses and pseudotypes**

Recombinant viruses were obtained by transfection of the appropriate virus vectors into LMH cells, except that supernatants were collected from day 3 to day 10 post transfection; virions were concentrated 20 to 50-fold by precipitation in 10% (w/v) PEG-8000 and 500 mM NaCl [2], resuspended in PBS with 10% (v/v) glycerol and stored at -80°C. For pseudotypes, cells were transfected with equal amounts of a vector encoding the env^-^ viral genome and vectors for the appropriate PreS/S proteins plus a vector for DHBV or HHBV S protein to ensure proper S expression regardless of potential S promoter mutations in the chimeras. Viral titers, as vge, were determined by DNA dot blot or by qPCR. Multiplicity of infection (MOI) was calculated assuming, operationally, that one vge equals one infectious particle.

**Quantitative PCR (qPCR)**

Quantitative PCR determinations were performed using a LightCycler 1.5 instrument (Roche) and SYBR Green (ABgene). DNase I treated cytoplasmic extracts and DNase I plus Pronase E digested culture supernatants (see above) served as templates for intracellular replicative intermediates (RIs) and secreted virion genomes, respectively. Intracellular DNA levels represent maximal values due to the presence of residual RC DNA from the inoculum; based on Southern blot results, the contribution of this input DNA was significant only at early timepoints for poorly PDH infectious samples such as wt-HHBV. Primer pairs usually used were the avihepadnavirus consensus primers D(+)1389 (TGGATTTCTCTCAGTTCTCC AAAGG) and D(-)1496 (TCCTGGGCATCCCCACGGC); to distinguish between DHBVm1 and Du-He4 in in vivo samples by genotype-specific PCR, the selective primers m1(+)877Spe (GgAGacTagTCCCAAAAGGGACTTTG) and He4(+)877Spe (GcAGac tagTCCCAcccgaattttcg) together with consensus primer D(-)1320Sal/Spe (TTAGGtCgaCTAGTATTCCCCCGAAGG) were employed. As established using defined mixtures of plasmid DNAs, under the conditions used each assay discriminated against the other genotype by at least five orders of magnitude. Cell numbers were determined based on a previously reported procedure [14] via quantification of the duck IFNα gene (Genbank accession: X84764) [15], calculated to be present in 10 copies per duck cell haploid genome [14], using primers IF1Xba (GATATCTAGaacgacacgcagcaagc) and IR1Sal/Spe (TTAGGtCGACTAGTAgg aggaagtgttggatgc), and assuming diploidy of the isolated hepatocytes [16] and a female ZW karyotype [17]. Plasmid contamination in DNA from transfected LMH cells was determined by quantitation of the *bla* gene present on all pCD16-based virus expression plasmids, using primers pBR/AmpR(+) (GCGAGACCCACGCTCACCGGC) and pBR/AmpR(-) (ACTTGGTTGAGTACTCACC), and exceeded in no case about 1% of the number of viral genomes measured.

**In vitro infections**

PDH were prepared as previously described [18]. For infection, cells were incubated overnight at the desired MOI with recombinant virus after overnight incubation with DNase I to remove residual plasmid DNA, or with the appropriate amount of viremic duck serum. For the HHBV pseudotype infection experiments, the inoculation period was extended to three days for maximal infection efficiency. Cells were harvested at the indicated time points, and total DNA including cccDNA was prepared by SDS lysis, pronase E digestion and extraction with phenol and chloroform as reported [19]. Viral genomes in secreted enveloped particles were quantitated by qPCR after DNase plus pronase E treatment and normalized to cell numbers as described above.

**In vivo infections**

Two to three day old Pekin ducklings were obtained from a commercial breeder, and held under veterinary supervision and according to German animal rights legislation. Preinoculation sera were tested by an anti-PreS immunodot blot using mAb 4F8 for congenital DHBV infection (found in 10 to 20% of the animals). Animals were injected into the foot vein with the indicated amounts of the recombinant virus preparations or viremic serum. Antigenemia was monitored by Western blotting for PreS/S using mAb 4F8, viremia by qPCR as described above for cell culture supernatants. To determine the kinetics of viral spread in the liver, one or two animals each were sacrificed at the indicated time points, and liver DNA was isolated using a commercial kit (DNeasy Blood and Tissue kit, Qiagen). For Southern blots, 10 µg of total liver DNA per lane was used; blots were hybridized with the bispecific probe and signals were quantified by phosphorimaging.

**Neutralization assay**

1ml of Du-He4 or DHBVm1 containing serum adjusted to 10^7^ vge/ml was incubated for one h with an equal volume of pooled anti-D-PreS/S sera [20], then 200 µl of the mixture (equivalent to 10^6^ vge) were injected per duckling. For the controls, the antiserum was replaced by normal duck serum. Infection was monitored over 28 d p.i. by immunodotblot for serum PreS using mAb 4F8, and by quantitation of viral loads by qPCR (Figure S4).

**References:**

1. Mandart E, Kay A, Galibert F (1984) Nucleotide sequence of a cloned duck hepatitis B virus genome: comparison with woodchuck and human hepatitis B virus sequences. J Virol 49: 782-792.

2. Protzer U, Nassal M, Chiang PW, Kirschfink M, Schaller H (1999) Interferon gene transfer by a hepatitis B virus vector efficiently suppresses wild-type virus infection. Proc Natl Acad Sci USA 96: 10818-10823.

3. Summers J, Smith PM, Huang MJ, Yu MS (1991) Morphogenetic and regulatory effects of mutations in the envelope proteins of an avian hepadnavirus. J Virol 65: 1310-1317.

4. Sprengel R, Kaleta EF, Will H (1988) Isolation and characterization of a hepatitis B virus endemic in herons. J Virol 62: 3832-3839.

5. Mueller-Hill K, Loeb DD (1996) Previously unsuspected cis-acting sequences for DNA replication revealed by characterization of a chimeric heron/duck hepatitis B virus. J Virol 70: 8310-8317.

6. Ishikawa T, Ganem D (1995) The pre-S domain of the large viral envelope protein determines host range in avian hepatitis B viruses. Proc Natl Acad Sci U S A 92: 6259-6263.

7. Prassolov A, Hohenberg H, Kalinina T, Schneider C, Cova L, et al. (2003) New hepatitis B virus of cranes that has an unexpected broad host range. J Virol 77: 1964-1976.

8. Beterams G, Nassal M (2001) Significant interference with hepatitis B virus replication by a core-nuclease fusion protein. J Biol Chem 276: 8875-8883.

9. Hu K, Beck J, Nassal M (2004) SELEX-derived aptamers of the duck hepatitis B virus RNA encapsidation signal distinguish critical and non-critical residues for productive initiation of reverse transcription. Nucleic Acids Res 32: 4377-4389.

10. Vorreiter J, Leifer I, Rösler C, Jackevica L, Pumpens P, et al. (2007) Monoclonal antibodies providing topological information on the DHBV core protein and avihepadnaviral nucleocapsid structure. J Virol 81: 13230-13234.

11. Urban S, Schwarz C, Marx UC, Zentgraf H, Schaller H, et al. (2000) Receptor recognition by a hepatitis B virus reveals a novel mode of high affinity virus-receptor interaction. Embo J 19: 1217-1227.

12. Xu Z, Ou JH (2004) Endogenous polymerase assay for the analysis of hepatitis B virus in transgenic mice. Methods Mol Med 95: 295-302.

13. Lenhoff RJ, Summers J (1994) Coordinate regulation of replication and virus assembly by the large envelope protein of an avian hepadnavirus. J Virol 68: 4565-4571.

14. Zhang YY, Zhang BH, Theele D, Litwin S, Toll E, et al. (2003) Single-cell analysis of covalently closed circular DNA copy numbers in a hepadnavirus-infected liver. Proc Natl Acad Sci USA 100: 12372-12377.

15. Nanda I, Sick C, Munster U, Kaspers B, Schartl M, et al. (1998) Sex chromosome linkage of chicken and duck type I interferon genes: further evidence of evolutionary conservation of the Z chromosome in birds. Chromosoma 107: 204-210.

16. Turin F, Borel C, Benchaib M, Kay A, Jamard C, et al. (1996) n-Butyrate, a cell cycle blocker, inhibits early amplification of duck hepatitis B virus covalently closed circular DNA after in vitro infection of duck hepatocytes. J Virol 70: 2691-2696.

17. Ellegren H (2001) Hens, cocks and avian sex determination. A quest for genes on Z or W? EMBO Rep 2: 192-196.

18. Tuttleman JS, Pugh JC, Summers JW (1986) In vitro experimental infection of primary duck hepatocyte cultures with duck hepatitis B virus. J Virol 58: 17-25.

19. Summers J, Smith PM, Horwich AL (1990) Hepadnavirus envelope proteins regulate covalently closed circular DNA amplification. J Virol 64: 2819-2824.

20. Rollier C, Sunyach C, Barraud L, Madani N, Jamard C, et al. (1999) Protective and therapeutic effect of DNA-based immunization against hepadnavirus large envelope protein. Gastroenterology 116: 658-665.
