# Supplementary material for: Heterologous Replacement of the Supposed Host Determining Region of Avihepadnaviruses: High In Vivo Infectivity Despite Low Infectivity for Hepatocytes
Source: PLoS Pathog. 2008 Dec 5;4(12):e1000230. doi: 10.1371/journal.ppat.1000230 (PMC2585059; doi:10.1371/journal.ppat.1000230)
Supplement: Figure S1 — Genetic tags for discrimination of chimeric from wild-type viruses. A. Partial restriction maps of preS-derived PCR amplicons from the viruses used in this study. DHBV16 and DHBV3 are the predominant wild-type DHBV strains endemic in the US and Europe, respectively. The tagged DHBV16 derivative DHBVm1, used as reference wild-type virus, contains the artificially introduced Bsm BI site present in all chimeras, and the Mro I site present in Du-He3 and Du-He4. B. Genotyping of selected chimeras after in vitro infection of PDH. The example shows the restriction patterns of PCR amplicons obtained from intracellular nucleocapsid-associated DNA of PDH inoculated with the indicated recombinant viruses. Each amplicon produced the expected unique pattern. The same type of analysis was used to confirm infection by the desired virus variants in vivo and to exclude congenital infection by DHBV3 or contamination with the laboratory strain DHBV16. (0.64 MB PDF) [file ppat.1000230.s002.pdf]

Supporting Figure S1

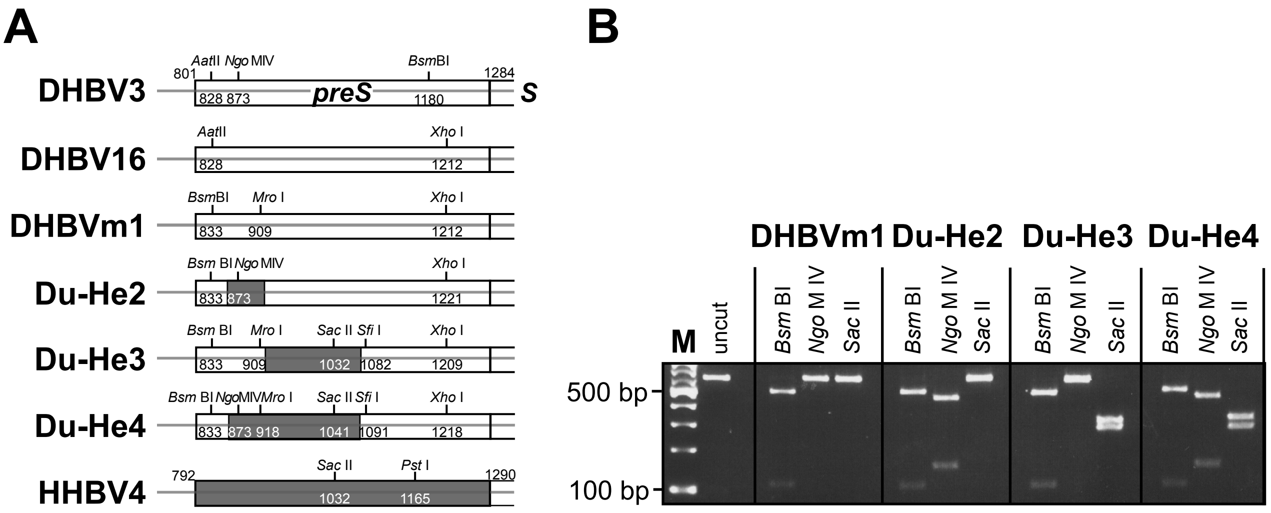

Figure S1. Genetic tags for discrimination of chimeric from wild-type viruses. A. Partial restriction maps of preS-derived PCR amplicons from the viruses used in this study. DHBV16 and DHBV3 are the predominant wild-type DHBV strains endemic in the US and Europe, respectively. The tagged DHBV16 derivative DHBVm1, used as reference wild-type virus, contains the artificially introduced Bsm BI site present in all chimeras, and the Mro I site present in Du-He3 and Du-He4. **B. Genotyping of selected chimeras after in vitro infection of PDH.** The example shows the restriction patterns of PCR amplicons obtained from total intracellular DNA of PDH harvested 15 days post inoculation with the indicated recombinant viruses. Each amplicon produced the expected unique pattern. The same type of analysis was used to confirm infection by the desired virus variants in vivo and to exclude congenital infection by DHBV3 or contamination with the laboratory strain DHBV16.
