# Supplementary material for: Heterologous Replacement of the Supposed Host Determining Region of Avihepadnaviruses: High In Vivo Infectivity Despite Low Infectivity for Hepatocytes
Source: PLoS Pathog. 2008 Dec 5;4(12):e1000230. doi: 10.1371/journal.ppat.1000230 (PMC2585059; doi:10.1371/journal.ppat.1000230)
Supplement: Figure S2 — Genetic tags are preserved upon in vivo infection of ducks with chimeras Du-He2, Du-He3 and Du-He4. A. Serum samples (day 7 p.i.) from ducks inoculated with the indicated recombinant viruses. PCR amplicons were incubated with the indicated restriction enzymes and the products were analyzed by agarose gel electrophoresis. B. Serum sample from vertical transmission experiment (embryo #e1). All samples produced input-virus specific fragment patterns (see Figure S1). (0.99 MB PDF) [file ppat.1000230.s003.pdf]

Supporting Figure S2

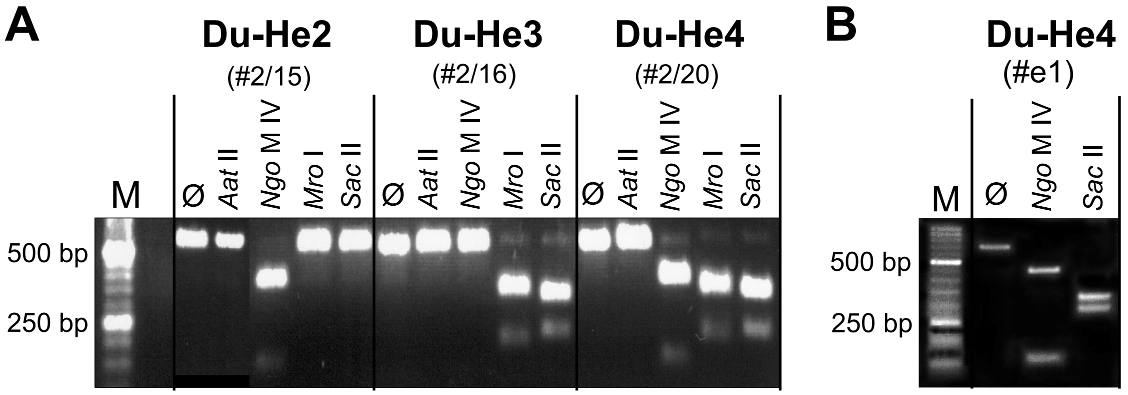

Figure S2. Genetic tags are preserved upon in vivo infection of ducks with chimeras Du-He2, Du-He3 and Du-He4. A. Serum samples (day 7 p.i.) from ducks inoculated with the indicated recombinant viruses. PCR amplicons were incubated with the indicated restriction enzymes and the products were analyzed by agarose gel electrophoresis. **B. Serum sample from vertical transmission experiment (embryo #e1).** All samples produced input-virus specific fragment patterns (see Figure S1).
