# Supplementary material for: Heterologous Replacement of the Supposed Host Determining Region of Avihepadnaviruses: High In Vivo Infectivity Despite Low Infectivity for Hepatocytes
Source: PLoS Pathog. 2008 Dec 5;4(12):e1000230. doi: 10.1371/journal.ppat.1000230 (PMC2585059; doi:10.1371/journal.ppat.1000230)
Supplement: Figure S3 — In vivo spread of Du-He4 is as fast as, or faster than that of wild-type DHBV. Ten ducklings each were inoculated with serum-derived DHBVm1 (from animal #4/6) or Du-He4 (from animal #4/17) and analyzed as described in the legend to Figure 8. Animal #9/9 from the DHBVm1 group showed no signs of productive infection and was excluded from further analyis. A. Kinetics of viremia. Serum samples from the indicated animals were collected at the indicated days p.i. and analyzed by DNA dot blot using a 32P labeled bispecific DNA probe; one out of several exposures is shown. The day 2 sample from animal #9/18 could not be analyzed. B. DNA dot blot of DHBV plasmid DNA standard used for calibration. A dilution series of plasmid pCD16 DNA containing the indicated amounts of viral genome equivalents was dotted on a membrane and detected with the identical probe as the dot blots shown in A. A graphic representation of the resulting values is shown in Figure 8B. (0.41 MB PDF) [file ppat.1000230.s004.pdf]

Supporting Figure S3

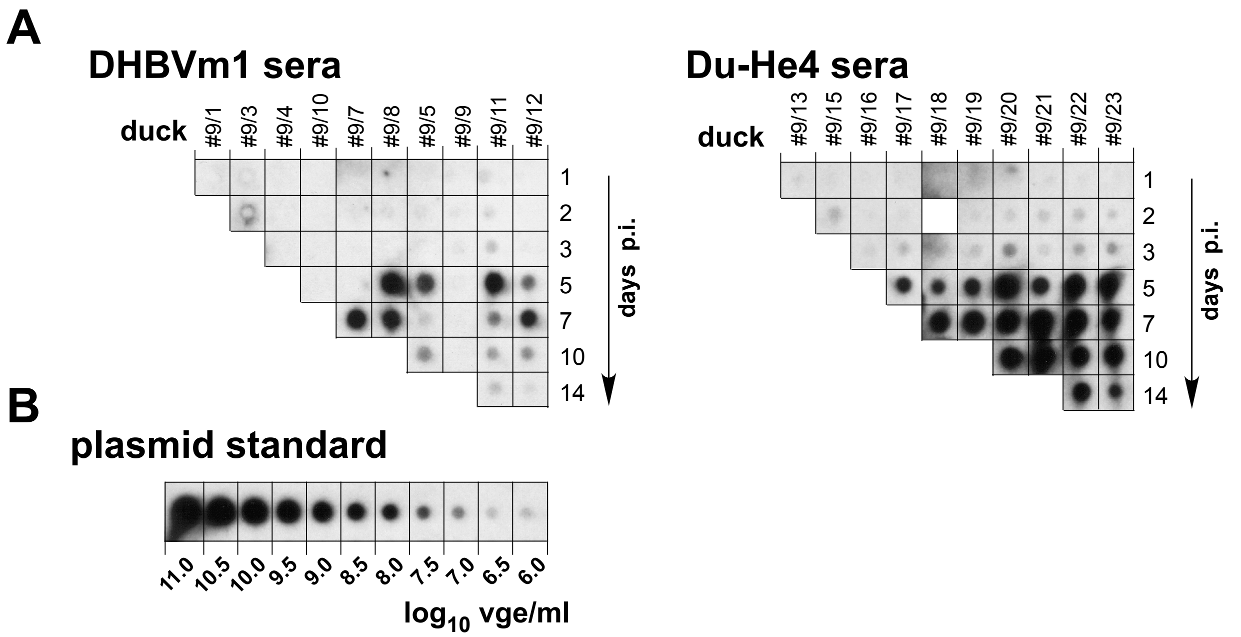

Figure S3. In vivo spread of Du-He4 is as fast as that of wild-type DHBV. Ten ducklings each were inoculated with serum-derived DHBVm1 (from animal #4/6) or Du-He4 (from animal #4/17) and analyzed as described in the legend to Figure 8. Animal #9/9 from the DHBVm1 group showed no signs of productive infection and was excluded from further analyis. **A. Kinetics of viremia.** Serum samples from the indicated animals were collected at the indicated days p.i. and analyzed by DNA dot blot using a ^{32}P labeled bispecific DNA probe; one out of several exposures is shown. The day 2 sample from animal #9/18 could not be analyzed. **B. DNA dot blot of DHBV plasmid DNA standard used for calibration.** A dilution series of plasmid pCD16 DNA containing the indicated amounts of viral genome equivalents was dotted on a membrane and detected with the identical probe as the dot blots shown in A. A graphic representation of the resulting values is shown in Figure 8B.
