# Supplementary material for: Heterologous Replacement of the Supposed Host Determining Region of Avihepadnaviruses: High In Vivo Infectivity Despite Low Infectivity for Hepatocytes
Source: PLoS Pathog. 2008 Dec 5;4(12):e1000230. doi: 10.1371/journal.ppat.1000230 (PMC2585059; doi:10.1371/journal.ppat.1000230)
Supplement: Figure S4 — Du-He4 is neutralized by duck sera against wild-type DHBV L protein. Serum samples containing 107 vge/ml of Du-He4 (from animal #4/17) or DHBVm1 (from animal #4/6) were incubated with an equal volume of a previously characterized DHBV neutralizing duck antiserum (α-DPreS/S), or with normal duck serum (NDS), for 1 h at room temperature. Aliquots containing 106 vge were inoculated into two ducklings each (animals #7/1,2: Du-He4+α-DPreS/S; #7/3,4: Du-He4+NDS; #7/5,6: DHBVm1+α-DPreS/S; #7/7,8: DHBVm1+NDS). Viremia was monitored by qPCR for 28 d p.i.; values below 104 vge/ml are close to the detection limit and may not be very accurate. In animal #7/5 (DHBVm1+α-DPreS/S; not shown) development of viremia was delayed but an increase in viral load from 8.3×10^3^ vge/ml at d 7 p.i. to 6.1×10^6^ vge/ml at d 11 indicated partial protection. (0.06 MB PDF) [file ppat.1000230.s005.pdf]

Supporting Figure S4

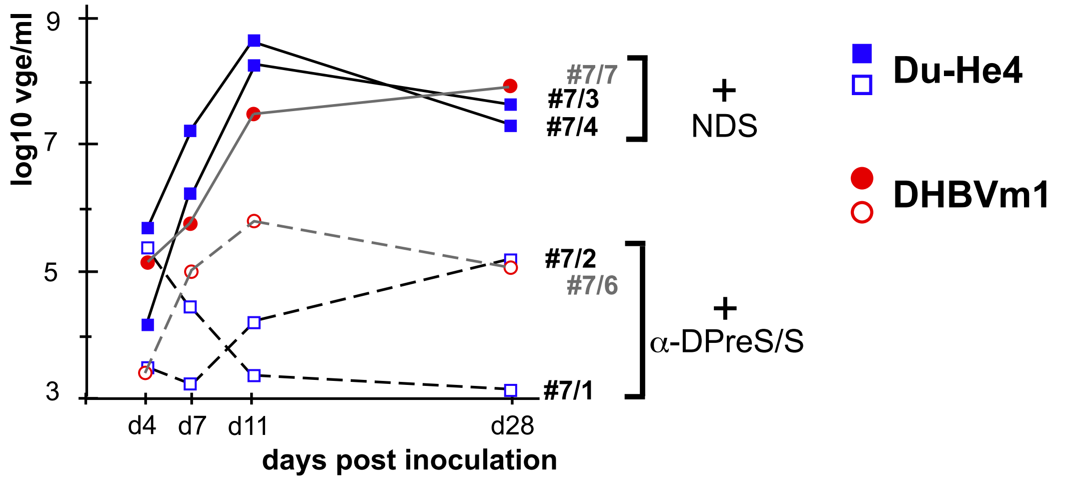

Figure S4. Du-He4 is neutralized by duck sera against wild-type DHBV L protein. Serum samples containing 10^7 vge/ml of Du-He4 (from animal #4/17) or DHBVm1 (from animal #4/6) were incubated with an equal volume of a previously characterized DHBV neutralizing duck antiserum (α-DPreS/S), or with normal duck serum (NDS), for 1 h at room temperature. Aliquots containing 10^6 vge were inoculated into two ducklings each (animals #7/1,2: Du-He4 + α-DPreS/S; #7/3,4: Du-He4 + NDS; #7/5,6: DHBVm1 + α-DPreS/S; #7/7,8: DHBVm1 + NDS). Viremia was monitored by qPCR for 28 d p.i.; values below 10^4 vge/ml are close to the detection limit and may not be very accurate. In animal #7/5 (not shown) an increase in viral load from 8.3 x 10^3 vge/ml at d 7 p.i. to 6.1 x 10^6 vge/ml at d 11 indicated partial protection.
